# Influence of premature rupture of membranes on the peripartum outcomes in vaginally intended breech deliveries at term: A FRABAT study

**DOI:** 10.1002/ijgo.70391

**Published:** 2025-07-23

**Authors:** Emmy Rasch, Anna Elisabeth Hentrich, Anne Kristina Kämpf, Eileen Deuster, Samira Catharina Hoock, Frank Louwen, Lukas Jennewein

**Affiliations:** ^1^ Department of Obstetrics and Perinatal Medicine University Hospital, Goethe University Frankfurt Frankfurt Germany

**Keywords:** breech delivery, prelabor rupture of membranes at term

## Abstract

**Objective:**

This large cohort study investigates the impact of premature rupture of membranes (PROM) on maternal and neonatal outcomes in breech deliveries. The aim is to contribute to a better understanding of vaginal breech delivery and its possible risk factors and to facilitate more informed decision‐making within the counseling process. This study compares perinatal outcomes in vaginally planned breech deliveries with and without PROM at term.

**Methods:**

A total of 2876 women with singleton breech presentation between 2007 and 2023 were included on this prospective cohort registry study. Among 1920 with intended vaginal delivery, 642 experienced PROM, while 1278 had spontaneous rupture of membranes (SROM). Of the women with PROM, 397 completed vaginal delivery, while the remainder underwent cesarean section after the onset of labor or after a PROM. Maternal and fetal outcome parameters were compared between groups using Pearson's *χ* ^2^‐test, the Kruskal–Wallis test, and multivariate regression analysis with Spearman's *ρ* correlation while a significance level of *P* < 0.05 applied.

**Results:**

No differences in maternal and fetal outcome were observed regarding the timing of membrane rupture in vaginally planned or completed breech deliveries at term. Fetal outcome parameters, including those measured by the modified PREMODA score, showed no significant difference in relation to intended vaginal breech delivery with PROM (PROM: 2.2%, SROM: 1.4%, *P* = 0.212). None of the maternal outcome parameters showed a significant difference between the two groups. A significant association was found between an increased maternal body mass index and the prevalence of PROM in breech presentation (PROM: 23.5 ± 4.2 kg/m^2^, SROM: 22.9 ± 3.7 kg/m^2^, *P* = 0.007). There was an increased rate of epidural anesthesia in the PROM group compared to the SROM group (PROM: 307 [57.6%], SROM: 661 [51.7%], *P* = 0.014).

**Conclusion:**

This study supports the proposition that PROM has little to no impact on the fetal and maternal outcome of breech deliveries. With appropriate expertise, based on these data, PROM cannot be considered a risk factor for adverse outcome in vaginal breech delivery. It should be noted that women delivering vaginally with a breech presentation and PROM at term might have a higher need for epidural anesthesia. Clinical management of cases with PROM can follow that of pregnancies with cephalic presentation.

## 1 INTRODUCTION

Various national guidelines suggest that vaginal delivery of a baby in breech presentation is a safe mode of birth.^1^, ^2^ However, due to often only partially evidence‐based inclusion and exclusion criteria for vaginal breech delivery, there is significant global uncertainty regarding the recommended mode of delivery.^3^, ^4^, ^5^, ^6^, ^7^ Because the prevalence of and thereby the experience in breech presentation is quite low and evidence‐based recommendations on delivery management are scarce, cesarean sections are performed for breech presentations in the majority of obstetrical centers.^3^, ^5^, ^6^, ^7^ This directly leads to an increase in cesarean‐related complications. These complications can include increased maternal morbidity, such as infections, thromboembolic events, and longer recovery times, as well as risks for future pregnancies, like placenta previa or accreta spectrum.^1^, ^8^ To optimize counseling on the mode of delivery and peripartum management, more research on vaginally planned breech deliveries is needed.

Regardless of the fetal presentation, premature rupture of membranes at term (PROM) occur in 8%–10% of cases.^9^ This refers to a gestational age of >37 weeks where the amniotic sac ruptures before the onset of labor contractions. In current studies and guidelines, PROM is associated with increased morbidity for both the mother and the child.^4^, ^10^, ^11^, ^12^ This is especially the case if delivery is not planned and initiated within 24 h, with guidelines recommending active management.^1^, ^2^, ^11^ Commonly studied criteria include the elevated risk of chorioamnionitis, endometritis, or confirmed or suspected neonatal sepsis in cases of PROM after 37 weeks.^10^, ^11^

In daily clinical practice, deliveries initiated by a rupture of the membranes and not with contractions can result in a complicated course of labor. The amniotic sac has a protective feature against pressure caused by the labor contractions, resulting in lower maternal pain levels and, in theory, fewer cases with a non‐reassuring fetal heart rate. Often, a change in pain levels in terms of pressure intensity and a change in fetal heart rate patterns occur when the amniotic sac ruptures during labor.^13^, ^14^ In breech presentation and cases with leading small fetal parts (complete breech, incomplete breech, and footling breech presentation), with ruptured membranes there is a higher probability of fetal foot or leg or even umbilical cord prolapse.^3^, ^15^ In obstetrical centers without a robust routine for managing labor in cases with breech presentation, a high need for security might lead to cesarean section recommendations because of those hypothesized risks even if no complication is present. This cautious approach, while aiming to ensure maternal and neonatal safety, might inadvertently contribute to the global trend of rising cesarean section rates.

The evidence regarding term PROM in pregnancies with breech presentation is very limited. A labor with PROM if often complicated because of anticipated umbilical cord prolapse and the risk of peripartum infection. This study investigates whether labor in term breech presentations carries a higher risk of complications when it begins with ruptured membranes compared to when the amniotic sac remains intact. By evaluating maternal and neonatal outcomes in breech presentations with PROM at term versus spontaneous labor at term, this research aims to contribute to the development of evidence‐based guidelines for managing breech deliveries with PROM.

## 2 MATERIALS AND METHODS

### 2.1 Patient cohort and patient selection

From 2007 to 2023, we conducted a prospective cohort registry study including all women who presented with a term fetus (>37 weeks) in breech presentation at the University Hospital in Frankfurt. The hospital's ethics committee provided consent for this study (2021–126). The study is registered in the German clinical study registry (DRKS00025030). Written consent for participation in the study was obtained from all participants. All data is analyzed after anonymization. A PROM at term is defined as rupture of the amniotic sac before the onset of labor.^16^ If labor does not begin, an antibiotic prophylaxis is offered after 12 h. Birth induction is offered after 24 h, which is in line with the German recommendations on PROM in cephalic presentation.

Exclusion criteria for a vaginal birth approach in women with breech presentation were estimated birth weight below 2500 g, uterine malformations (e.g., bicornuate uterus), uterine fibromas suspected to complicate the course of delivery, an intertuberous distance of below 11 cm (measured with magnetic resonance imaging) and/or clinical examination in primiparous women, fetal malformations suspected to complicate the course of delivery, and signs of diabetic fetopathy. Protocols for vaginal breech delivery management have been previously published. In the second stage of labor, women are in an upright position. In contrast to a supine position, manual assistance is not necessary on a routine basis. In case of a delayed delivery because of impacted arms/shoulders, the Louwen maneuver is performed. To assist head delivery, the Fank Nudge is utilized. Both maneuvers are carried out while the mother stays in an upright position.^3^

**FIGURE 1:**
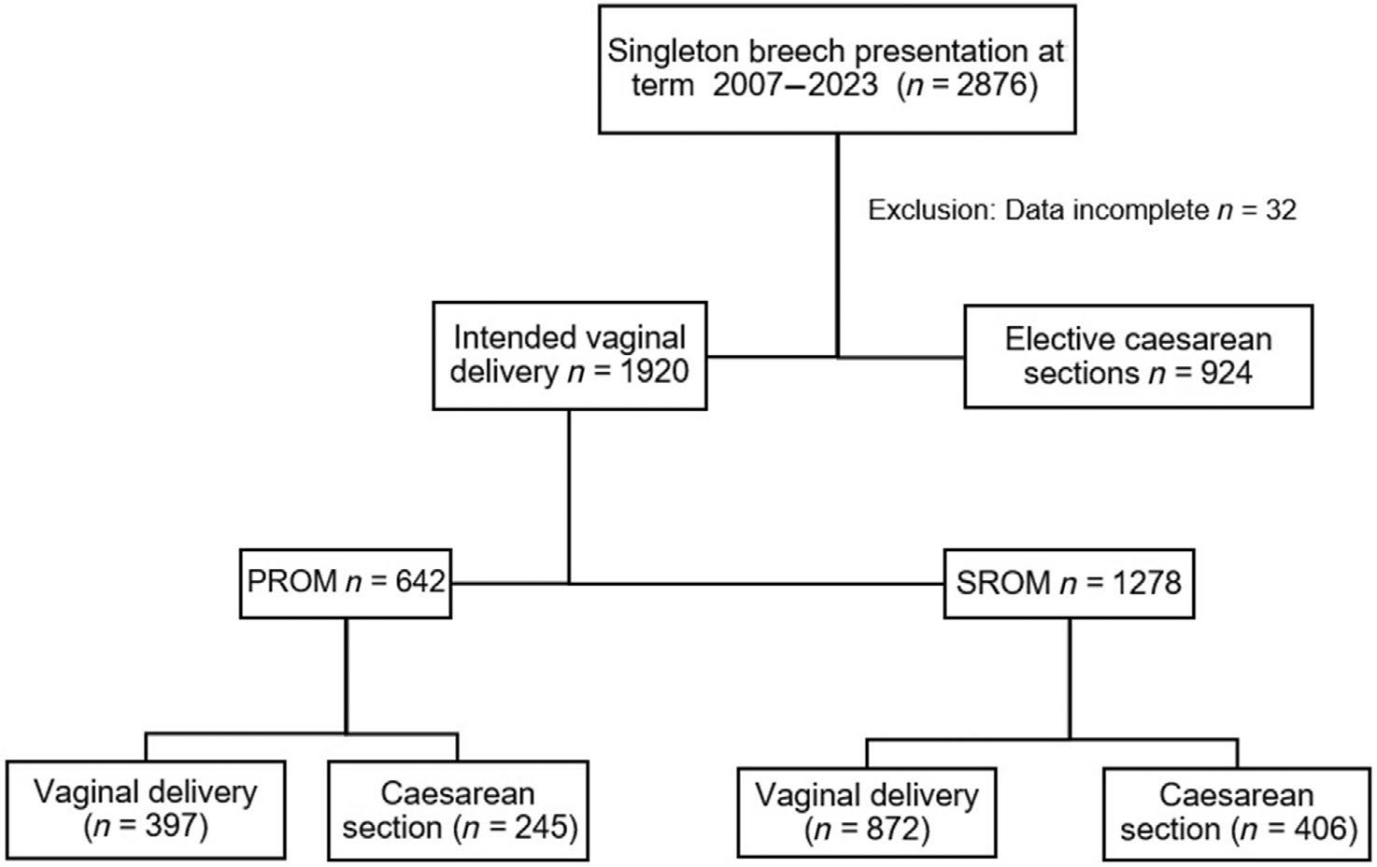
Study cohort flow chart. PROM, premature rupture of membranes; SROM, spontaneous rupture of membranes.

Of the 2876 Women presenting with a fetus in breech presentation, 1952 opted for a vaginal delivery. Thirty‐two cases had to be excluded because of incomplete data. All women were counseled on their preferred birth mode. Advantages and possible complications of both a vaginal birth approach and elective cesarean section were explained to all patients during counseling. An external cephalic version was offered to all women. After PROM, an antibiotic prophylaxis was offered to all women after 1920 vaginally attempted deliveries (Figure 1). Of the 1920 intended vaginal deliveries, 642 women experienced PROM. In this subcohort, 397 deliveries proceeded vaginally, while 245 cases required a cesarean section.

The university hospital's patient management system was used for data acquisition after the patients had been discharged.

### 2.2 Statistical analyses

Variables were tested if a normal distribution was applied using the Kolmogorov–Smirnov test. Parametric variables were compared for group differences with two‐sided Student's *t*‐testing. Non‐parametrical variables were analyzed with Pearson's *χ* ^2^‐test and Fischer's exact test. Non‐parametric multivariate regression analysis was performed using Spearman's *ρ* multivariate correlation. Statistical analyzes were conducted using JMP 18 software (SAS Institute, Cary, USA).

### 2.3 Outcome parameters

The primary outcome measure was fetal morbidity. We used a modified PREMODA score, adapted from the PREMODA study.^6^ A case counted as a delivery with short‐term morbidity if one or more of the following features applied: 5‐min APGAR <4, intubation >24 h, stay at the neonatal intensive care unit (NICU) >4 days, and neurological deficit at discharge. Each case with a positive PREMODA score was reviewed in detail, and cases with morbidity without a logical clinical association with the delivery mode were excluded (e.g., postpartum infection, hyperbilirubinaemia, and birth defects).

## 3 RESULTS

A total of 1920 women aiming for a singleton vaginal breech delivery presented at the University Hospital Frankfurt between 2007 and 2023. In 642 cases (33.4%), PROM occurred, whereas in 1278 cases (66.6%) membranes ruptured spontaneously after the onset of labor (SROM). Age, height, weight, and parity were equally distributed between the two groups (see Table 1). In cases of PROM, the mode of delivery was vaginal delivery in 397 cases (61.8%), including manual assistance. Cesarean section was performed in 245 cases (38.2%) compared to 405 cases in the SROM group (31.7%). The characteristics analyzed for perinatal maternal outcome, such as blood loss, postpartum hemorrhage, and signs of infection, showed similar results for both groups. The body mass index (BMI) measured before pregnancy was found to be significantly higher in the PROM group (PROM: 23.5 kg/m^2^, SROM: 22.9 kg/m^2^, *P* = 0.007, Table 1). The gestational duration was significantly shorter in cases with PROM compared to the SROM group (PROM: 277.2 ± 8.5 days, SROM: 280.0 ± 8.0 days, *P* < 0.0001, Table 1). Postpartum hemoglobin levels were significantly lower in the PROM group (PROM: 10.7 g/dL, SROM: 10.9 g/dL, *P* = 0.040, Table 1). The rate of cesarean sections was significantly higher in the PROM group (PROM: 254 [38.2%], SROM: 405 [31.7%], *P* = 0.0047, Table 1). The rate of epidural analgesia in the PROM Group was significantly higher, with an increase of nearly six percentage points compared to the control group (PROM: 370 [57.6%], SROM: 661 [51.7%], *P* = 0.014, Table 1).

**TABLE 1:** Parameters of maternal outcome in vaginal intended deliveries in breech position.

Parameter	PROM *n* = 642	SROM *n* = 1278	*P*‐value
Nulliparous women [*n* (%)]	392 (61.1%)	722 (56.5%)	0.056
Age (M ± SD; years)	32.6 ± 4.3	32.4 ± 4.3	0.270
Height (M ± SD; cm)	168.3 ± 6.1	168.9 ± 6.1	0.054
Weight (M ± SD; kg)	66.7 ± 12.9	65.4 ± 11.2	0.110
BMI (M ± SD; kg/kg/m^2^)	23.5 ± 4.2	22.9 ± 3.7	0.007*
Pregnancy duration (M ± SD; days)	277.2 ± 8.5	280.0 ± 8.0	<0.0001*
Complete breech [*n* (%)]	413 (64.7%)	827 (64.7%)	0.870
Cesarean section [*n* (%)]	254 (38.2%)	405 (31.7%)	0.0047*
Epidural analgesia [*n* (%)]	370 (57.6%)	661 (51.7%)	0.014*
Signs of infection during birth [*n* (%)]	13 (2.0%)	41 (3.2%)	0.139
Postpartum hemorrhage [*n* (%)]	15 (2.3%)	36 (2.8%)	0.537
Systemic pre‐existing conditions (e.g., hypertension) [*n* (%)]	128 (19.9%)	236 (18.5%)	0.438
Hypertension in pregnancy, preeclampsia [*n* (%)]	8 (1.3%)	13 (1.0%)	0.649
Blood coagulation disorders [*n* (%)]	16 (2.5%)	37 (2.9%)	0.611
Blood loss during birth (M ± SD in mL)	340.2 ± 8.4	341.2 ± 236.6	0.195
Labor induction [*n* (%)]	118 (18.4%)	226 (17.7%)	0.708
Hb at hospital Discharge (M ± SD; g/dL)	10.7 ± 1.4	10.9 ± 1.4	0.040*

* Significance level is reached with *p* < 0.005.

Abbreviations: BMI, body mass index; M, mean; PROM, premature rupture of membranes; SD, standard deviation.

The fetal outcome parameters, including infections, neurological deficits, intubation, birth injuries, adaptation disorders, NICU stay >4 days, the 5‐min APGAR score, and base excess individually showed no significant differences between intended vaginal deliveries with PROM versus SROM (Table 2). No significant increase in umbilical cord prolapse could be demonstrated in the PROM group compared to the control group (PROM: 84 [13.1%], SROM: 149 [11%], *P* = 0.17, Table 2). A modified PREMODA score was used, combining different morbidity parameters. No significant differences in neonatal morbidity potentially associated with the timing of membrane rupture were observed (PROM: 14 [2.2%], SROM: 18 [1.4%], *P* = 0.212, Table 2). Neonatal infection rates were not significantly different between groups (PROM: 28 [4.4%]; SROM: 46 [3.6%], *P* = 0.413). Significant differences were observed in birth length (PROM: 52.1 ± 2.7 cm, SROM: 52.4 ± 2.6 cm, *P* = 0.0.028, Table 2), weight (PROM: 3303.3 ± 377.3 g, SROM: 3383.6 ± 429.8 g, *P* = 0.0002, Table 2), and head circumference (PROM: 35.3 ± 1.3 cm, SROM: 35.6 ± 1.4 cm, *P* < 0.0001, Table 2), with all three parameters lower in the PROM group compared to the control group.

**TABLE 2:** Parameters of fetal outcome in intended vaginal deliveries in breech position.

Parameter	PROM *n* = 642	SROM *n* = 1278	*P*‐value
Birth weight (M ± SD; in g)	3303.6 ± 377.3	3383.6 ± 429.8	0.0002*
Head circumference (M ± SD; in cm)	35.3 ± 1.3	35.6 ± 1.4	<0.0001*
Birth length (M ± SD; in cm)	52.1 ± 2.7	52.4 ± 2.6	0.028*
Base excess child (M ± SD)	−5.3 ± 4.1	−5.5 ± 3.9	0.262
5‐min APGAR <4 [*n* (%)]	3 (0.5%)	8 (0.6%)	0.664
NICU >4 days [*n* (%)]	39 (6.1%)	53 (4.2%)	0.062
Transfer to a pediatric ward [*n* (%)]	53 (8.3%)	87 (6.8%)	0.250
Umbilical cord complication [*n* (%)]	84 (13.1%)	140 (11.0%)	0.170
Transient adjustment disorder [*n* (%)]	31 (4.8%)	68 (5.3%)	0.646
Birth injuries child [*n* (%)]	4 (0.6%)	8 (0.6%)	0.994
Intubation child [*n* (%)]	9 (1.4%)	9 (0.7%)	0.135
Neurological deficits [n (%)]	4 (0.6%)	5 (0.4%)	0.483
Neonatal infection [*n* (%)]	28 (4.4%)	46 (3.6%)	0.413
Gestational diabetes, insulin [*n* (%)]	18 (2.8%)	25 (2.0%)	0.236
PREMODA [*n* (%)]	43 (6.7%)	61 (4.8%)	0.079
PREMODA rel.t.Dev‐Mode [*n* (%)]	14 (2.2%)	18 (1.4%)	0.212

* Significance level is reached with *p* < 0.005.

Abbreviations: M, mean; NICU, neonatal intensive care unit; PROM, premature rupture of membranes; SD, standard deviation.

A sub cohort of vaginal deliveries after exclusion of cases with cesarean section was analyzed. Primiparity, delivery positions, and manual assistance were not significantly different between vaginal PROM and vaginal SROM subcohorts. The PROM group did not exhibit an increased prevalence of fetuses in the Frank breech position (PROM: 269 [67.8%], SROM: 583 [66.8%], *P* = 0.748, Table 3). Among women who delivered vaginally, no significant differences were observed in the rate of epidural anesthesia. Regarding maternal outcome parameters like second‐ and third‐degree perineal tears (PROM: 10 [2.5%], SROM: 12 [1.4%], *P* = 0.146, Table 3) and blood loss (PROM: 292.8 ± 214 mL, SROM: 295.9 ± 219 mL, *P* = 0.890, Table 3), no significant differences were found. As with the intended vaginal births, Hb levels at discharge were found to be significantly lower in cases of PROM (PROM: 10.8 ± 1.4 g/dL, SROM: 11.0 ± 1.5 g/dL, *P* = 0.018, Table 3).

**TABLE 3:** Parameters of maternal and fetal outcome in vaginally completed deliveries in breech position.

Maternal parameters	PROM (vaginal) *n* = 397	SROM (vaginal) *n* = 873	*P*‐value
Nulliparous women [*n* (%)]	208 (52.4%)	414 (47.4%)	0.101
Assisting manual maneuvers [*n* (%)]	169 (42.6%)	389 (44.6%)	0.508
Assisted arm delivery [*n* (%)]	99 (24.9%)	204 (23.4%)	0.543
Assisted head delivery [*n* (%)]	152 (38.3%)	339 (38.8%)	0.854
Duration of opening period (M ± SD in minutes)	333.6 ± 317.3	296.6 ± 257.5	0.447
Duration of pressing (M ± SD in minutes)	50.2 ± 62.2	40.3 ± 51.3	0.018*
Frank breech [*n* (%)]	269 (67.8%)	583 (66.8%)	0.748
Perineal injury [*n* (%)]	200 (50.5%)	398 (45.7%)	0.115
Perineal tear III + IV [*n* (%)]	10 (2.5%)	12 (1.4%)	0.146
Epidural analgesia [*n* (%)]	210 (52.9%)	416 (47.7%)	0.083
Blood loss during birth (M ± SD in mL)	292.8 ± 214	295.9 ± 219.1	0.890
Labor induction [*n* (%)]	55 (13.9%)	113 (12.9%)	0.657
Hb at hospital discharge (M ± SD; g/dL)	10.8 ± 1.4	11.0 ± 1.5	0.018*
Fetal parameters			
Base excess (M ± SD)	−6.8 ± 4.0	−6.8 ± 3.6	0.629
5 min APGAR <4 [*n* (%)]	3 (0.8%)	7 (0.8%)	0.931
NICU >4 days [*n* (%)]	20 (5.0%)	31 (3.6%)	0.211
Transfer to a pediatric ward [*n* (%)]	30 (7.6%)	52 (6.0%)	0.282
Transient adjustment disorder [*n* (%)]	20 (5.0%)	42 (4.8%)	0.862
Birth injuries, hematoma [*n* (%)]	4 (1.0%)	8 (0.9%)	0.876
Intubation [*n* (%)]	6 (1.5%)	8 (0.9%)	0.347
Neurological deficits [*n* (%)]	4 (1.0%)	2 (0.2%)	0.061
Neonatal infection [*n* (%)]	13 (3.3%)	23 (2.6%)	0.524
PREMODA [*n* (%)]	23 (5.8%)	37 (4.2%)	0.226
PREMODA rel.t.Dev‐Mode [*n* (%)]	12 (3.0%)	14 (1.6%)	0.098

* Significance level is reached with *p* < 0.005.

Abbreviations: M, mean; NICU, neonatal intensive care unit; PROM, premature rupture of membranes; SD, standard deviation.

For fetal parameters, including the modified PREMODA score (PROM: 12 [3%], SROM: 14 [1.6%], *P* = 0.098, Table 3), no significant differences in outcomes were observed between the two groups. Other characteristics such as neonatal neurological deficits (PROM: 4 [1.0%], SROM: 2 [0.2%], *P* = 0.061, Table 3), intubation rates (PROM: 6 [1.5%], SROM: 8 [0.9%], *P* = 0.347, Table 3), or birth injuries (PROM: 4 [1.0%], SROM: 8 [0.9%], *P* = 0.876, Table 3) were not found to be significantly higher in the PROM group.

In the multivariate analysis of various parameters in women who completed vaginal delivery from a breech presentation with PROM, three independent significant associations were identified. Women with PROM who required labor induction showed a significant association with a longer interval between membrane rupture and delivery (Spearman *ρ* = 0.3317, *P* < 0.001, Table 4). Higher fetal birth weight was associated significantly with a higher preconceptional BMI of the birthing woman (Spearman *ρ* = 0.1196, *P* = 0.018, Table 4) and with a significantly higher rate of labor inductions (Spearman *ρ* = 0.1072, *P* = 0.034).

**TABLE 4:** Multivariate regression analysis of women with vaginal delivery and PROM.

Variable	With variable	Spearman *ρ*	*P*‐value
Blood loss	Duration from membrane rupture to delivery (min)	0.0629	0.241
PREMODA rel.t.Dev‐Mode	Duration from membrane rupture to delivery (min)	0.0450	0.385
PREMODA rel.t.Dev‐Mode	Blood loss	0.0630	0.228
Preconceptional BMI	Duration from membrane rupture to delivery (min)	0.0789	0.131
Preconceptional BMI	Blood loss	−0.0602	0.252
Preconceptional BMI	PREMODA rel.t.Dev‐Mode	0.0569	0.262
Induction (0 = no, 1 = yes)	Duration from membrane rupture to delivery (min)	0.3317	<0.0001*
Induction (0 = no, 1 = yes)	Blood loss	0.1246	0.0166*
Induction (0 = no, 1 = yes)	PREMODA rel.t.Dev‐Mode	−0.0282	0.575
Induction (0 = no, 1 = yes)	Preconceptional BMI	0.0848	0.094
Birth weight	Duration from membrane rupture to delivery (min)	0.0138	0.791
Birth weight	Blood loss	0.0823	0.115
Birth weight	PREMODA rel.t.Dev‐Mode	0.0356	0.479
Birth weight	Preconceptional BMI	0.1196	0.0181*
Birth weight	Induction (0 = no, 1 = yes)	0.1072	0.0327*
Perineal tear III + IV	Duration from membrane rupture to delivery (min)	0.0187	0.719
Perineal tear III + IV	Blood loss	0.0592	0.257
Perineal tear III + IV	PREMODA rel.t.Dev‐Mode	0.0654	0.194
Perineal tear III + IV	Preconceptional BMI	−0.0714	0.160
Perineal tear III + IV	Induction (0 = no, 1 = yes)	−0.0646	0.199
Perineal tear III + IV	Birth weight	0.0031	0.951

* Significance level is reached with *p* < 0.005.

Abbreviations: BMI, body mass index; PROM, premature rupture of membranes.

No association was found between higher‐degree perineal tears and an extended interval between membrane rupture and delivery, blood loss, preconceptional BMI, induction, or fetal birth weight in women who delivered vaginally from a breech presentation with PROM. No enhanced neonatal outcomes, as measured by the modified PREMODA score, were associated significantly with any of the assessed parameters, such as birth weight, labor induction, or preconceptional BMI.

## 4 DISCUSSION

According to various guidelines, vaginal delivery out of breech presentation is a safe option. Reduction of unnecessary cesarean sections might be a way to reduce perinatal morbidity and mortality in newborns and mothers because high cesarean section rates worldwide are associated with adverse outcome.^1^, ^2^, ^3^, ^4^, ^5^, ^6^, ^7^, ^17^

A study conducted by Louwen et al. in 2017 demonstrated that serious fetal and neonatal morbidity potentially associated with the mode of delivery is generally low at our center and comparable between upright vaginal breech births and planned cesarean sections (odds ratio 1.37, 95% confidence interval 0.10–19.11).^18^ These favorable outcomes might, among other factors, be attributable to the use of the upright birthing position in our clinical practice.

The rupture of membranes before labor can affect the delivery outcome. In this study, the influence of a PROM\ at term and its impact on peripartum outcome is investigated for the first time in a large cohort (of 1920 deliveries).

Fetal short‐term morbidity was not associated with PROM in our analysis. Understanding that PROM does not worsen outcomes in vaginal breech deliveries could help address uncertainties in this area and support offering vaginal delivery as a viable option. It also provides evidence‐based certainty in labor management. Importantly, umbilical cord prolapse was not reported significantly more often after PROM in breech delivery, contradicting common supposition.

Regarding the maternal parameters, obesity is widely acknowledged as a significant risk factor for dystocia during labor.^19^, ^20^ Current literature commonly applies the WHO classification of obesity, which defines it as a BMI of 30 kg/m^2^ or higher. Among the complications associated with maternal obesity are preterm labor and PROM.^21^ With our data, we can confirm the association of overweight and obesity with the incidence of PROM in term delivery. The relationship between PROM and maternal obesity remains the focus of ongoing research and appears to be a complex, multifactorial process. Proposed contributing factors include hormonal imbalances, increased inflammatory activity, and alterations in connective tissue, all of which may compromise the integrity of the fetal membranes, potentially leading to premature rupture.^22^

In our study, which examined both planned and completed vaginal breech deliveries with PROM, a higher maternal BMI was found to be associated with an increased incidence of PROM. This finding is consistent with previous research, such as a 2021 study by Sunarno et al.^12^ Furthermore, other studies have demonstrated that, even in cases of elevated BMI, vaginal breech delivery does not result in increased maternal or neonatal morbidity compared to cesarean delivery, provided that the patient cohort is carefully selected, and the mother delivers in an upright position.^23^

In our study, the rate of cesarean sections was significantly higher when PROM occurred (PROM: 254 [38.2%], SROM: 405 [31.7%], *P* = 0.0047, Table 1). This finding concurs with data from current guidelines, where PROM is considered an indication for labor induction or cesarean section, particularly when labor does not commence within 24 h after membrane rupture.^1^, ^2^ In this way, signs of infection can lead to indications for a cesarean section, while birth induction is also associated with a higher probability of a cesarean delivery.^24^ Several studies have demonstrated that delayed labor onset beyond 24 h is associated with higher infection rates, leading to worse outcomes for both mother and child.^10^, ^11^

Regardless of the timing of membrane rupture, a study by Allert et al. (2023) found no significant association between the use of epidural “walking” anesthesia in vaginal breech deliveries and increased cesarean section rates, nor with increased fetal or neonatal morbidity. However, a significant association was established between epidural anesthesia and prolonged first and second stages of labor, as well as an increased need for manual assistance during breech deliveries.^25^

In our study cohort, epidural analgesia in breech delivery was found to be associated with PROM when compared to women with SROM (Table 1). The significantly higher demand for epidural anesthesia might suggest that PROM increases the pain intensity during labor. When the amniotic fluid is gone, the pressure of fetal parts on the lower soft tissue and the pelvic inlet could lead to an enhanced pain experience, especially if there is an extended latency phase. Further investigation into this aspect could provide additional insights into pain management in such cases. The reasons for this phenomenon are the focus of current research, which explores the potential role of prostaglandins in this mechanism.

Prostaglandins play a central role in pregnancy, the initiation of labor, and the overall birth process.^26^ Their functions are complex and diverse and remain a subject of ongoing research. Among their key roles are the inflammatory processes, pain mediation, the enhancement of uterine contractility, and cervical ripening. Their production during pregnancy is mainly in the fetal membranes. Prostaglandins such as PGE2 and PGF2α appear to be particularly significant in cervical ripening and the onset of labor, as their cervical receptors double in number during the labor process.^27^ As outlined, PROM results from a multifactorial process, leading to a scenario in which amniotic fluid prostaglandins could encounter an immature cervix with insufficient receptor availability, resulting in inadequate cervical dilation. Given their broader physiological roles beyond labor, prostaglandins may also contribute to increased maternal pain due to inflammation and higher uterine contractability, thereby potentially leading to a higher demand for analgesia. These insights underline the complex interplay between hormonal dynamics and labor mechanisms, highlighting prostaglandins as a key factor in ongoing research into labor induction and management.

Regarding fetal parameters, it is important to note that no significant difference was observed in infection‐related parameters between the PROM group and the control group. However, other studies suggest that PROM increases the risk of fetal infection, particularly with prolonged intervals between PROM and the onset of labor, as well as with an increased number of vaginal examinations.^2^, ^10^, ^11^ Of course, active management and surveillance to detect signs of infection (analogous to cephalic deliveries) are recommended. In our study center, labor management regarding the time and presence of ruptured membranes were handled equivalently to cephalic deliveries. For example, a prophylactic antibiotic treatment was applied in cases of detected group B streptococcus or absence of contractions after PROM for 12 h. It is possible that this guideline‐based treatment of PROM could have contributed to overall low infection rates.^2^

A previous study conducted at our center in 2019 examined the incidence of umbilical cord loops and prolapses in frank breech and complete breech presentations. It was found that these occurred significantly more often when the baby was in the complete breech presentation compared to the frank breech position, while there were no significant differences in fetal short‐term morbidity.^3^ In this context, it is interesting to note that, according to our data, prelabor rupture of membranes did not significantly increase the risk of umbilical cord prolapse (see Table 2). This suggests that umbilical cord prolapse is more likely influenced by fetal presentation and the fetal leg posture than by the timing of membrane rupture.

Other examined parameters that could potentially influence fetal outcomes also showed no significant differences in the PROM group, both in intended vaginal deliveries and those that were completed vaginally. The slightly shorter gestational duration in the planned vaginal breech deliveries with PROM could, in turn, explain the somewhat lower birth weight, size, and head circumference.

Concerning the results of the multivariate regression analysis, a significant association was identified between labor induction and the interval from membrane rupture to delivery. This association could be explained by the fact that, in our clinic, labor induction is offered after 24 h and recommended after 48 h in cases of PROM, thereby linking longer latency periods to the need for induction.^2^, ^10^, ^11^ Further, in the multivariate analysis, a significant correlation was identified between higher maternal BMI and both increased neonatal birth weight and an elevated likelihood of labor induction. This finding aligns with prior evidence indicating that elevated maternal BMI is associated with greater fetal growth and an increased need for medical interventions during childbirth.^28^

A study with a design similar to ours was conducted by Cattin et al. in 2016, which produced consistent findings but with a significantly smaller sample size (*N* = 209). The results of Cattin et al. align with ours, showing that neonatal parameters, such as the 5‐min APGAR score, also demonstrated no significant differences with respect to the timing of membrane rupture. The 2016 study also reported a slightly shorter duration of pregnancy in cases of PROM, while both studies observed a significantly higher cesarean section rate during labor in the PROM group. As in our study, no significant difference was found in infection parameters, such as chorioamnionitis or Group B streptococcal infection.^4^

A limitation of our collected data is its unicentered design. A multicenter study could validate the results and contribute to advancing knowledge on this topic. Further, this study did not include long‐term data to assess the prolonged effects on both mother and child. As a result, potential late‐onset complications or long‐term consequences remain unexamined and may require further research.

This study provides evidence to challenge the cautious approach toward vaginal breech deliveries with PROM. With appropriate counseling and an upright birth position, PROM does not adversely affect fetal or maternal outcomes in vaginally attempted breech delivery. Our findings are critical for preparing medical centers to manage this type of delivery effectively and safely and should contribute to generate guidelines and practice aids on the subject.

## AUTHOR CONTRIBUTIONS

Emmy Rasch: conceptualization, data curation, investigation, methodology, writing – original draft, visualization, project administration. Lukas Jennewein: conceptualization, methodology, supervision, writing – original draft, writing – review and editing, resources. Frank Louwen: supervision, writing – review and editing. Anna Elisabeth Hentrich, Anne Kristina Kämpf, Eileen Deuster, Samira Catharina Hoock: data curation, writing – review and editing. All authors have reviewed and approved the final version of the manuscript.

## FUNDING INFORMATION

None.

## CONFLICT OF INTEREST STATEMENT

The authors have no conflicts of interest to declare.

## Data Availability

Data is shared upon request. Other publications of our FRABAT cohort, using the same data of this prospective register study have published available public datasets.
